# Endocrine Disruption at the Androgen Receptor: Employing Molecular Dynamics and Docking for Improved Virtual Screening and Toxicity Prediction

**DOI:** 10.3390/ijms19061784

**Published:** 2018-06-15

**Authors:** Joel Wahl, Martin Smieško

**Affiliations:** Molecular Modeling, Department of Pharmaceutical Sciences, University of Basel, Klingelbergstrasse 50, CH-4056 Basel, Switzerland; joel.wahl@unibas.ch

**Keywords:** androgen receptor, ensemble docking, virtual screening, molecular dynamics simulations, agonists, antagonists

## Abstract

The androgen receptor (AR) is a key target for the development of drugs targeting hormone-dependent prostate cancer, but has also an important role in endocrine disruption. Reliable prediction of the binding of ligands towards the AR is therefore of great relevance. Molecular docking is a powerful computational method for exploring small-ligand binding to proteins. It can be applied for virtual screening experiments but also for predicting molecular initiating events in toxicology. However, in case of AR, there is no antagonist-bound crystal structure yet available. Our study demonstrates that molecular docking approaches are not able to satisfactorily screen for AR antagonists because of this reason. Therefore, we applied Molecular Dynamics simulations to generate antagonist AR structures and showed that this leads to a vast improvement for the docking of AR antagonists. We benchmarked the ability of these antagonist AR structures discriminate between AR antagonists and decoys using an ensemble docking approach and obtained promising results with good enrichment. However, distinguishing AR antagonists from agonists with high confidence is not possible with the current approach alone.

## 1. Introduction

The androgen receptor (AR) is the main biomolecular target involved in the development and progression of the hormone-dependent prostate cancer [1]. Furthermore, disruption of the androgen system is associated with decreased sperm count, increased infertility [2], and diabetes mellitus [3]. Therefore, in silico evaluation of small-molecule binding at the AR is of high relevance for the design of novel antiandrogens for the treatment of prostate cancer [1,4,5,6], but also for the screening of potential androgen-disrupting chemicals [7] causing endocrine disorders [8,9]. Several in silico toxicity prediction models and tools have shown their potential to identify endocrine disrupting chemicals [10,11,12].

One major challenge associated with the computational prediction of binding modes and affinities of compounds towards the androgen receptor by structure-based methods is the lack of a published structure of antagonist-bound AR and experimental and theoretical studies aimed at elucidating the mechanisms of AR antagonism [13,14,15,16,17,18,19,20].

In its unbound state, the AR is complexed with heat-shock and chaperone proteins that stabilize the *apo* state of the protein [4]. Agonist binding triggers and stabilizes a conformational change that facilitates coactivator binding to a so-called activation function-2 (AF2) region [4]. A widely proposed mechanism for AR antagonism is the displacement of the Helix-12 (H12) upon ligand binding, leading to distortions in the AF2 site, preventing coactivator binding [13]. This mechanism was recently supported by computational studies employing Molecular Dynamics (MD) simulations [14,15,16]. Furthermore, the repositioning of H12 was confirmed to be the mechanism of antagonism for the estrogen receptor (ER) and the peroxisome proliferator-activated receptor (PPAR) [21].

Additional insights into to structural determinants for androgen antagonism were gained by structure-activity relationship studies, where the systematic influence of scaffold change on androgen agonism or antagonism was investigated [18,19,20]. Bohl and coworkers resolved the crystal structures for several steroidal agonists and steroidal and nonsteroidal antagonists towards the wild type (WT) AR and T877A and W741L mutants. They showed how the LBD of the AR can accommodate the B-ring of agonist S-1 by the repositioning of the indole ring of Trp741 [18]. McGinley and coworkers developed pan-antagonists by increasing the size of the B-ring [20]. This was based on the hypothesis that the increased bulkiness of the ring results in displacement of H12. This hypothesis was later disproved by the publication of a crystal structure of a naphthyl-containing WT agonist bound to the T877A form of the androgen receptor [19]. The receptor could accommodate the bulky ring substituent by a rearrangement of the Met745 side chain.

Regarding B-ring containing AR binders, the chemical nature of the linker seems to be the determining factor in converting agonists to antagonist and vice versa. The antagonist behavior of bicalutamide versus the agonist behavior of compound of S-22, an AR agonist containing an oxygen linker, can be explained by the presence of the much bulkier sulfonyl linker in the former [19].

Molecular Dynamics simulations (MD simulations) were applied in various studies to investigate structural determinants of androgen receptor antagonism [14,15,16,17]. Zhou and coworkers could show by analyzing replica exchange molecular dynamics simulations (REMD) that AR antagonism correlates with structural fluctuations of helix 12 [17]. Duan et al. applied microsecond long MD simulations and enhanced sampling to reveal the structural dynamics of HFT and bicalutamide binding to AR and also observed increased fluctuations for helix 12 [14]. Liu and coworkers simulated bicalutamide binding towards androgen WT and several mutations and identified a steric clash with helix 12 as the structural determinant for WT AR antagonism [15]. These studies established the applicability of molecular simulations for gaining structural insights into AR antagonism.

However, MD simulations of ligand-receptor complexes need a valid input structure of the bound ligand in the binding site and furthermore, due to their computational expense, cannot be applied to large libraries of compounds that are usually used in virtual screening campaigns. Structure-based virtual screening (SBVS) [22,23] based on molecular docking [24] can bridge this gap by generating conformations of the bound ligand (“poses”) at the binding site of a receptor, the docking poses are accompanied by a score which is an estimate of the affinity of the scored ligand towards the receptor. Ligand enrichment is then based on the docking scores. SBVS is now a widely used method in the field and has displayed several success stories in hit identification [22,25].

Furthermore, molecular docking can be applied to toxicology profiling of chemical compounds by assessing how well a chemical entity fits into the binding site [7,26]. Molecular docking studies require a representative receptor conformation for the prediction of binding poses and binding affinities of the screened ligands. This structure can for example be obtained by protein crystallography. For the AR, where a crystal structure in the antagonist conformation is not available, this is not feasible. By only applying docking to the agonist-bound structure, the outcomes of the virtual screening campaigns are expected to be biased towards compounds that act in an agonistic manner. One possibility to create an antagonistic structure of the AR is to apply homology modeling [27,28,29], taking experimental antagonistic structures of related Nuclear Receptors (NR) such as the estrogen receptor (ER). Whereas this strategy led to the successful discovery of novel antiandrogens, the docking accuracy of these antagonist structures were not evaluated.

Bisson et al. used a biased probability Monte Carlo procedure and iteratively refined receptor conformations that are biased to yield good discrimination of agonists and antagonists [30]. We used a different approach and applied MD simulations of various antagonists bound to WT AR. We used representative structures of these simulations (obtained by clustering of the trajectories) in an ensemble docking approach and validated if this strategy can be used for improved screening for AR antagonists, an application that is highly relevant for drug discovery and toxicology studies. In an ensemble docking approach, flexible ligand—rigid protein (the degrees of freedom of the receptor are not altered) docking is applied to different protein conformations obtained by crystal structures or from a Molecular Dynamics trajectory [31,32,33].

To validate the ability of MD simulations to generate antagonist-conformations of the AR, we ran several simulations of antagonist-bound WT AR and used a clustering approach to generate a structural ensemble for docking towards the AR. We then docked androgen antagonists towards this antagonist-ensemble and calculated the performance metrics compared to a set of decoys. Furthermore, we also constructed an agonist ensemble by simulating agonist-bound AR complexes and to assess if the performance for agonist screening is improved compared to the ensemble obtained from crystal structures.

A generally similar strategy was applied by Swift and coworkers [34] where AR conformations were generated by MD simulations and the best scoring ensembles in discriminating actives and decoys based on the ROC AUC values were identified by different search methods. However, they only run a simulation of one receptor-ligand complex and furthermore, no distinction between agonists and antagonists was made.

All evaluations of the docking accuracy were done target the wild type AR, but the strategy can be extended to include AR mutants as well.

The agonist and antagonist structural ensemble presented in our study have the potential to improve virtual screening campaigns and toxicology predictions for AR antagonism.

## 2. Results

### 2.1. Docking of AR Agonists, Antagonists and Decoys towards Experimental AR Structures

A diverse ensemble (by binding site shape similarity, see Methods Section) of five experimental AR structures (PDB IDs: 3B66, 3G0W, 3V49, 4HLW, and 2PNU) was used for an initial evaluation of the docking performance of Glide SP at the AR (crystal structures ensemble, Table 1).

As a first step, we conducted a cross-docking experiment, where we docked all five ligands from the crystal structures into all five receptor conformations to see if the Glide-SP score assigns the best score for a ligand to the corresponding structure it was extracted from. We determined furthermore how well the experimental pose is reproduced in terms of RMSD (self-docking).

**Figure 1:**
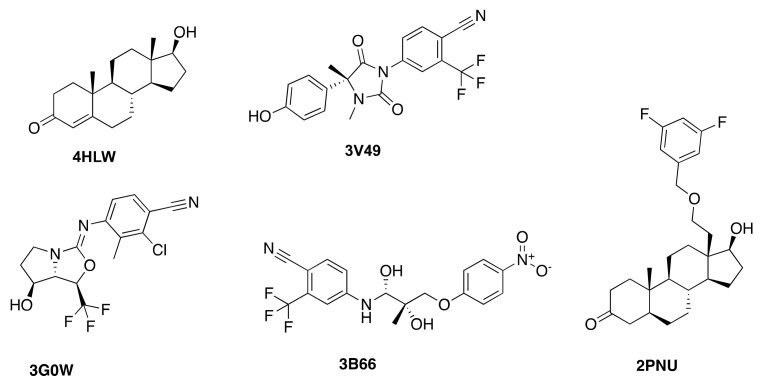
2D Structures of androgen receptor (AR) agonists bound to the five AR crystal structures.

All ligands showed the highest predicted affinity towards their corresponding native (experimental) protein conformation in the cross-docking experiment (Table 1). Furthermore, all native poses were recovered with satisfying accuracy in the self-docking experiments (RMSD values for self-docking: 3B66: 0.22 Å, 2PNU: 0.25 Å, 3G0W: 0.18 Å, 3V49: 0.49 Å, 4HLW: 0.80 Å). The most promiscuous conformation is 2PNU, reporting Glide Scores for all five ligands that were docked. On the other hand, the native ligand of the structure 2PNU (EM5744) corresponds to a bulky ligand (Figure 1) that could only be successfully docked into its native protein conformation. Binding of EM5744 requires a substantial induced fit, induced by rearrangement of Met895 and Trp741. None of the four other protein structures display this structural feature and none of them can therefore accommodate this type of ligand. The magnitude of the induced fit is also expressed by significant differences in binding site volumes of the different structures (2PNU: 261 Å^3^, 3B66: 189 Å^3^, 3G0W: 157 Å^3^, 3V49: 171 Å^3^, 4HLW: 168 Å^3^). The importance of the induced fit for binding at the AR was confirmed in a previous study [26].

In a next step, an agonist and antagonist set, taken from the NRlist BDB [35], and a set of 3000 decoys, taken from the enhanced directory of useful decoys (DUD-E) [36] were docked into the ensemble and docking performance was evaluated using the area under the receiver operating characteristic (AUC ROC). For the docking of the agonist versus the decoy set, Glide SP reached an impressive AUC ROC of 0.92 (Table 2), showing that discrimination between AR agonists and decoys is achieved to a high degree.

Taking experimentally validated decoys can be an alternative to the DUD-E, as described by Lagarde [37]. In case of the androgen receptor, the antagonist set can serve as a challenging decoy set. The ROC AUC in this case amounted to 0.80 (Table 2), showing discrimination between agonists and antagonists.

**Figure 2:**
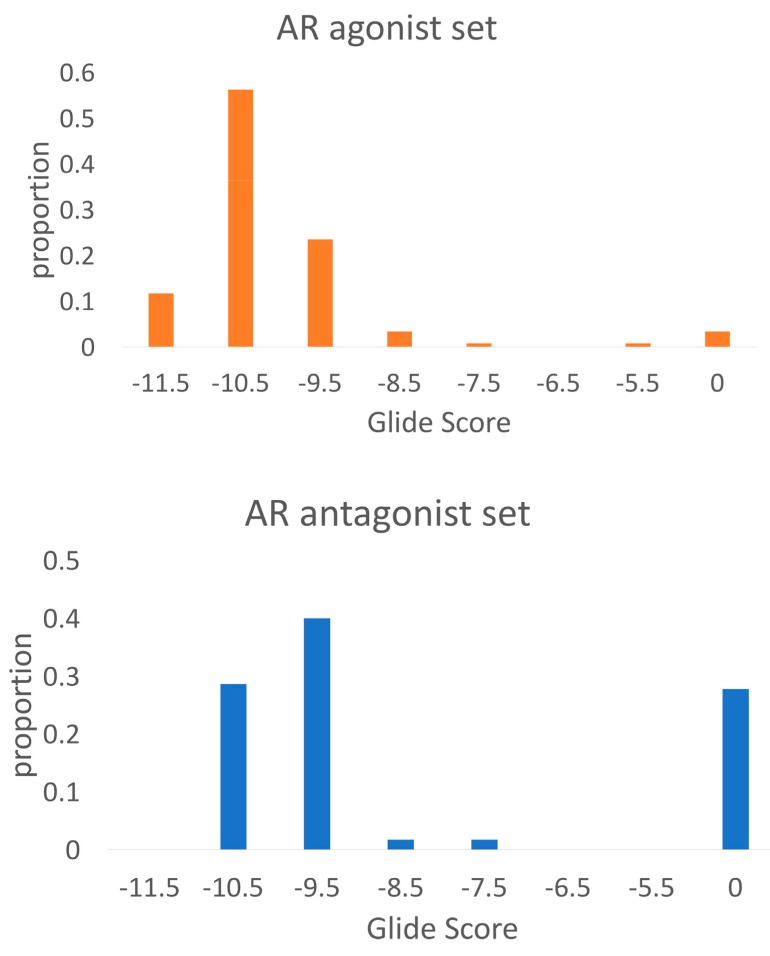
Glide docking score distributions for AR agonists and antagonists docked towards a structural ensemble of five agonist AR crystal structures. A docking score of 0 indicates that Glide could not successfully dock the ligand into any member of the ensemble.

The docking score distributions (Figure 2) for the AR agonists and antagonists show in general lower Glide SP Scores (corresponding to higher affinities) for the AR agonists. 68% of the agonists reached a docking score of −10.0 or lower, compared to 29% of the antagonists. Moreover, 32 out of 115 antagonists (28%) could not be successfully docked into any member of the ensemble, compared to 3 agonists were no docking solution was found.

As a third evaluation, we used the antagonist set as the active set and the DUD-E set as decoys. In this case, the ROC AUC reached 0.65 (Table 2). This shows that, whereas antagonists docked to the agonist structures of AR were ranked slightly higher than a set of decoys, they, in general, display a low affinity towards the agonist structures and in certain cases cannot fit into the binding site. As a consequence, structure-based virtual screenings or toxicology screenings based on agonist crystal structures are expected to be successful for the identification of AR agonists, but can only insufficiently model AR antagonists, and therefore, the need for AR structures that correspond to the antagonist conformation is high.

### 2.2. Generation of AR Antagonist Structures Using Flexible Docking, Binding Pose Metadynamics and Classical MD Simulations

**Figure 3:**
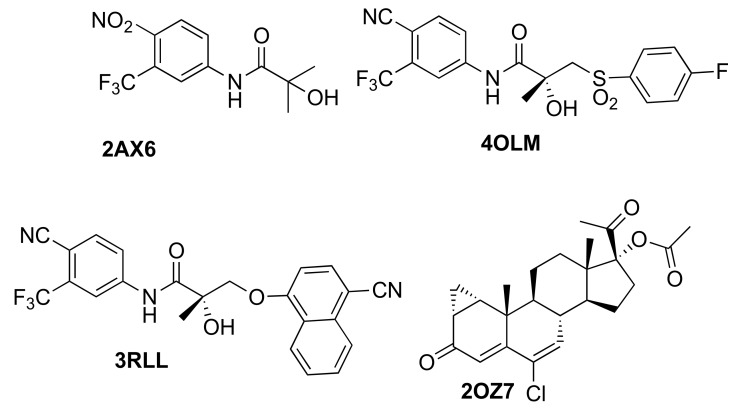
AR antagonists used for the generation of the ensemble of antagonist AR structures.

We used crystal structures of four different AR WT antagonists that act as agonists towards AR receptors with either a T877A or W741L mutation (PDB IDs: 2AX6 (T877A), 2OZ7 (T877A), 3RLL (T877A), 4OLM (W741L)). The antagonist ligand of 2AX6 [18] corresponds to Hydroxyflutamide (HFT), 4OLM [38] corresponds to R-Bicalutamide, and 2OZ7 [39] to cyproterone acetate. The ligand in the structure 3RLL [19] is named as structure 7 in the original publication and will be referred as Duke-7 in the following. Whereas 3 out of the 4 antagonists share the common scaffold from HFT, they differ in terms of presence and size of the B-ring (4OLM, 3RLL) (Figure 3) and the type of the linker. 2OZ7 has a steroid-core and is therefore structurally different.

We mutated the corresponding amino acids back to obtain the WT receptor. These structures will be termed as 2AXA_WT, 4OLM_WT, 3RLL_WT, and 2OZ7_WT in the following. We then employed induced fit docking (IFD) to dock the antagonists back into the corresponding WT structures and applied binding pose metadynamics [40] (see Methods Section) to assess the structural stability of the obtained binding poses. The most stable pose was then subject to a 100 ns MD simulation. From these trajectories, representative structures were extracted as the cluster centers of the three most populated clusters (see Methods Section). These representative structures then were used for the evaluation of the docking performance and for the selection of the best-performing AR antagonist ensemble (explained in the Methods Section).

**Figure 4:**
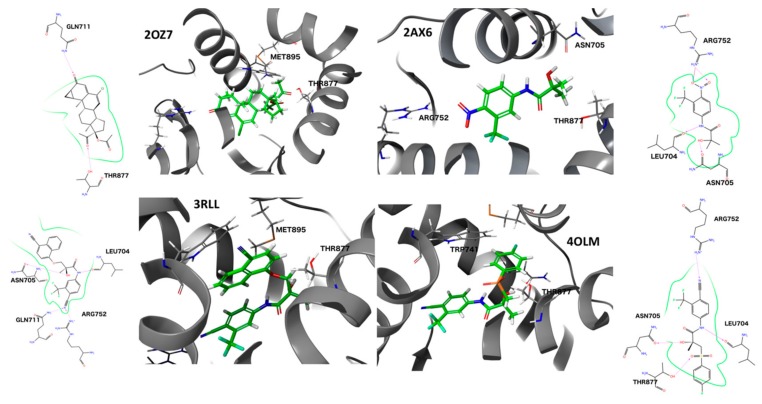
Docking poses of AR antagonists at AR wild type (WT) obtained from induced fit docking (IFD) and binding pose metadynamics.

For 2AX6_WT, the IFD protocol identified four distinct poses: The terminal hydroxyl group can have two different orientations (hydrogen bond either to Thr877 or Asn705). Furthermore, the phenyl ring can have two different orientations as well as the amide group. These four poses were then assessed by the binding pose metadynamics protocol and ranked by their corresponding PoseScores, which is an indication of the stability of the pose [40]. More details are given in the method section. The most stable pose (Figure 4) is characterized by the same binding motif that is present for HFT bound to AR T877A, exhibiting hydrogen bonding to of the ligand hydroxyl group to the side chain amide oxygen of Asn705.

The same protocol was applied to the other antagonist ligands. For 4OLM_WT, six different poses were identified by IFD, mainly differing by the orientation of the B-ring. The orientation of the B-ring in the most stable pose (Figure 4) differs vastly from the observed orientation in the crystal structure with the W741L mutation. The presence of the bulky tryptophan side chain makes the accommodation of the B-ring together with the bulky sulfonyl linker more demanding, explaining the occurrence of a different ring- and linker orientation.

Four distinct poses were found for 3RLL_WT by IFD. The bulkiness of the B-ring doesn’t allow alternative orientations and the identified poses are differing in the orientation of the A-ring. The best-scored pose (Figure 4) has the identical binding motif as in the crystal structure bound to T877A AR.

For 2OZ7, seven poses could be identified by IFD, differing in the orientation of the terminal acetate and keto groups. Due to the presence of a hydrogen bond to Thr877, the orientation of the keto group for the identified pose at the WT AR differs from the one that is observed in the crystal structure at the T877A AR (Figure 4).

The structures with the most stable poses for all complexes were used as input structures for 100 ns MD simulations. We also conducted 100 ns MD simulations of the original PDB complexes (AR mutations) to get an ensemble of agonist structures for comparison and assessment of how the mutation affects the structural dynamics of the receptor.

**Figure 5:**
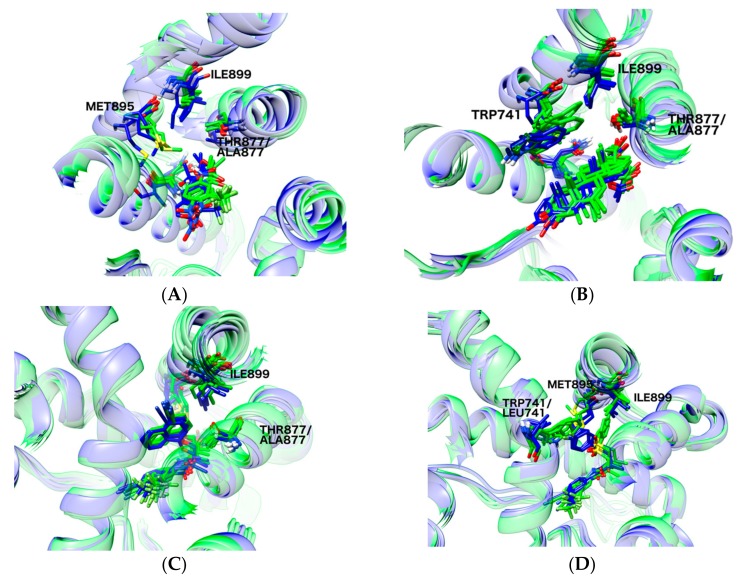
Cluster centroids from 100 ns MD simulation of four different ligands bound to mutated (agonist activity) and wild type (antagonist activity) AR. The structures from the mutated structures are depicted in blue, the WT structures in green. PDB IDs: (**A**) 2AX6; (**B**) 2OZ7; (**C**) 3RLL; (**D**) 4OLM.

A comparison of the representative structures (cluster centroids) from the 100 ns MD simulations started from the original PDB structures (agonist activity towards mutants) and from the stable docking poses from the IFD/binding pose metadynamics towards the WT (antagonist activity) is shown in Figure 5.

Structure 2AX6 corresponds to Hydroxyflutamide (HFT) bound to T877A AR. Towards this mutant, HFT acts as an agonist, whereas it has antagonist activity towards the WT AR. The terminal isopropanol group of HFT shows different orientations for the representative MD structures of the wild-type and the T877A structure (Figure 5, panel A). This is probably due to a smaller steric demand of the alanine side chain compared to threonine. This also leads to a different orientation of Ile899 and Met895. Furthermore, the backbone atoms of ILE899 are shifted away from the binding site for the WT structure.

For cyproterone acetate (CPA, Figure 5B), similar observations can be made. For the wild-type simulations, the backbone of Ile899 is shifted away from the binding site. This is accompanied by a shift of the ring moiety of Trp741. The keto group of CPA interacts with the Thr877 side chain in all snapshots for the WT simulation.

Ligand Duke-7 (Figure 5C) has a bulky B-ring with an oxygen linker. The presence of the bulkier Thr877 side chain in the wild-type forces a shift in the oxygen linker and again results in a slight movement of Ile899 away from the binding site.

The biggest structural differences between WT simulation and mutant simulation are observed for the binding of R-bicalutamide (Figure 5D). The W741L mutation can accommodate the B-ring with the bulky sulfonyl-linker, whereas for the wild type, there is a steric clash with the indole ring of Trp741, and the B-ring undergoes structural change and points away from the binding site, thereby pushing away the backbone atoms of Met895 and Ile899.

### 2.3. Docking Towards an Antagonist Ensemble Obtained from MD Simulations

The analysis of the antagonist-bound MD trajectories of the AR clearly indicated structural differences compared with trajectories of mutated AR, for which the antagonists are converted to agonists. However there remains the question if these antagonist-structures created by MD simulations can be used for improved prediction of AR antagonism by molecular docking.

As a first assessment, a set of antagonists and decoys was docked to the antagonist ensemble obtained from the MD simulations. An exhaustive search was then applied to pick a subset of the ensemble, consisting of five structures (see Methods Section). The number was chosen since it provides a good trade-off between covering the necessary conformational flexibility and still achieving results with little computational resources. Analyses with varying ensemble sizes confirmed this choice (results not shown). The ensemble was picked that obtained the best AUC ROC for discrimination between antagonists and agonists (ensemble MD_antagonist_1, Table 2).

The best ensemble reached a promising AUC-ROC of 0.86, indicating that indeed the antagonist structures created by MD simulations can be used for structure-based virtual screening of AR antagonists. The antagonist ensemble from the MD clearly outperforms the agonist ensemble from the crystal structure in terms of antagonist docking. Only one antagonist molecule could not be successfully docked into the ensemble, a vast improvement compared with 32 unsuccessful docking attempts from the agonist crystal ensemble. The best performing ensemble was composed of the structures 2AX6_WT_1, 2AX6_WT_2, 2OZ7_WT_1, 2OZ7_WT_2, 2OZ7_WT_3 (the final number refers to the index of the cluster centroid obtained from the clustering of the trajectories) and it will be termed as the MD_antagonist_1 ensemble in the following.

**Figure 6:**
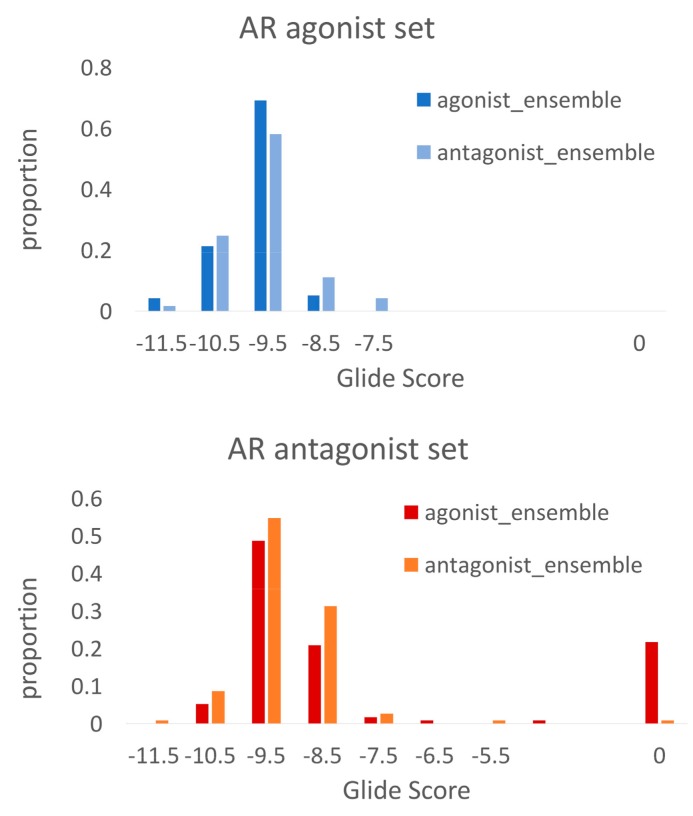
Docking score distributions for agonist and antagonist ligands docked to the agonistic and antagonistic receptor ensembles.

As a second evaluation, the agonist ligand set was docked towards the MD_antagonist_1 ensemble and the AUC ROC against the decoy set was determined. The obtained value of 0.92 indicates that this ensemble is not selective for AR antagonists, but also shows high promiscuity for the agonist ligands. This can be seen in Figure 6, where the distribution of the Glide scores of the agonists are similar for both docking at the agonist ensemble and at the antagonist ensemble.

### 2.4. Docking Towards an Agonist Ensemble Obtained from MD Simulations

**Figure 7:**
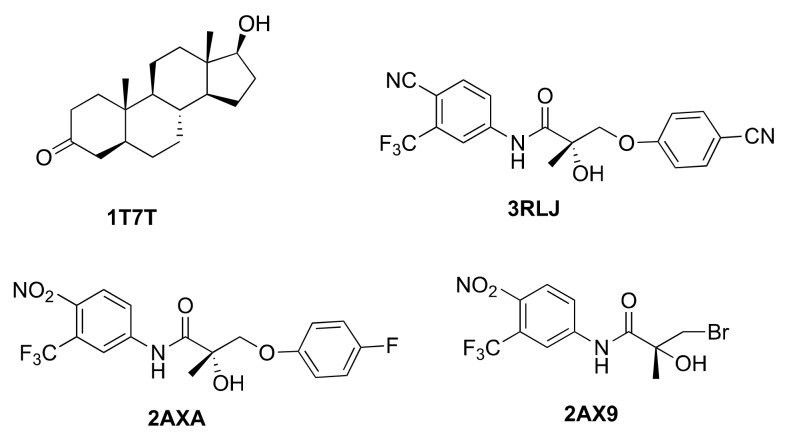
AR agonists used for the generation of the ensemble of agonist AR structures.

In order to have consistent structural ensembles for docking of both agonist and antagonist, we also conducted classical MD simulations of four structurally diverse agonists bound to WT AR (Figure 7). The input structures were directly taken as the crystal structures (PDB IDs: 1T7T, 2AXA, 2AX9, 3RLJ). We docked the agonist set to all 12 representative structures derived from clustering of the MD trajectories (four trajectories and three cluster centers each) and towards the same set of 3000 decoys as for the antagonist experiment. The best ensemble of five structures, showing the best discrimination between agonists and decoys, was again determined by an exhaustive search (ensemble MD_agonist_1). The best performing agonist ensemble reached an impressive AUC-ROC of 0.96 (Table 2) and consisted of the structures 1T7T_1, 1T7T_2, 2AX9_1, 2AX9_2, 3RLJ_1. All 117 agonist ligands could be successfully docked into the ensemble.

The antagonist ligands reached an AUC ROC of 0.69 (Table 2) at the MD_agonist_1 ensemble against the decoy set, implying that the selected ensemble is selective for agonists and shows low promiscuity for the antagonists. This is confirmed by Figure 6, where the Glide scores for the antagonists docked to the antagonist ensemble are, in general, more favorable than the scores at the agonist ensemble. Furthermore, 22 antagonists could not be successfully docked into the agonist ensemble.

In order to verify if the improved AUC ROC values for the MD-derived ensembles compared to the crystal structures really stem from the sampling of agonist and antagonist-like conformations and not just the better sampling of the receptor conformational space in general, we also determined the AUC ROC against the decoy set of docking the agonist ligands into the antagonist ensemble and vice versa by selecting the best performing agonist ensemble for the docking of the antagonists (ensemble MD_agonist_2) and the best performing antagonist ensemble for the docking of the agonists (ensemble MD_antagonist_2) (Table 2). The best performing agonist ensemble for the antagonist set had an AUC ROC of 0.74, significantly worse than the one of the best performing antagonist ensembles. On the other hand, the antagonist ensemble achieving the best AUC ROC for the docking of agonists reached an AUC ROC of 0.96, confirming that AR agonists exhibit good docking scores towards both the agonist as well as the antagonist ensemble.

### 2.5. Discriminating Agonists from Antagonists

The above results indicate that ensemble-docking towards MD-derived structures can be used to distinguish agonists from decoys and antagonists from decoys, which is definitely a step forward for structure-based design at the AR. Yet, there remains the question if this strategy can distinguish agonists from antagonists. Therefore, a new search for well-performing ensembles was conducted. For the selection of the antagonist ensemble, the objective function was the summed AUC-ROC for (i) docking the antagonist ligands and the DUD-E decoys and (ii) docking the antagonists and taking the agonists as decoys. The same search was performed for the agonist ensemble, where the agonists were docked and the AUC-ROC was evaluated with taking (i) the DUD-E decoys and (ii) the antagonists as decoys. The best antagonist ensemble with best discrimination against decoys and agonists had AUC-ROC values of 0.79 (decoys) and 0.56 (agonists) (ensemble MD_antagonists_3, Table 2). For the agonist ensemble, the AUC-ROC values were 0.96 (decoys) and 0.82 (antagonists) and it coincided with the best performing ensemble picked for maximum AUC-ROC against the decoys only (ensemble MD_agonist_1, Table 2). This ensemble facilitates discrimination of AR agonists against AR antagonists and decoys at the same time. For the antagonist structures, an ensemble that can successfully distinguish antagonists from decoys and agonists could not be found.

This implies that when we conduct a virtual screening campaign or toxicology screening using the presented agonistic ensemble, the compounds with the highest score are the agonist compounds and good selectivity over decoys and antagonists can be achieved. On the other hand, conducting the same screening using the antagonistic ensemble, agonists, and antagonists reach a similar score, making the identification of antagonists based on docking scores impossible.

This is confirmed by the docking score distributions (Figure 6).

### 2.6. Structural Comparison between Antagonist and Agonist Ensembles

**Figure 8:**
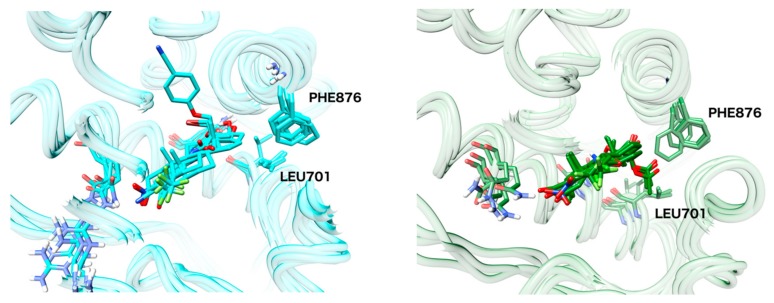
MD_agonist_ensemble_1 (**left**) and MD_antagonist_ensemble_1 (**right**).

The ensembles MD_antagonist_1 and MD_agonist_1 are depicted in Figure 8. Visual inspection surprisingly revealed only minor differences in the placement of helix12. The major differences are visible for the amino acids Phe876 and Leu701, exhibiting a much bigger conformation diversity for the antagonist ensemble.

The movement of Phe876 and Leu701 is most prominent for the member 2OZ7_WT_2 of the antagonist ensemble. The movement of these two amino acids facilitates an opening of the binding pocket and therefore, sterically demanding, large androgen antagonists with linear shapes can be docked to this structure, whereas docking them to any structure of the agonist ensemble is impossible and leads to steric clashes. Yet, it still remains unclear how this class of AR antagonists exhibits their mode of action if displacement of helix12 is not the underlying mechanism.

## 3. Materials and Methods

### 3.1. AR Agonists, Antagonists and Decoys

The androgen agonist and antagonist sets were downloaded from the NRlist BDB [35] (downloaded: September 2017). We manually curated some of the compounds by checking the primary literature sources of all agonists and antagonists with reported *K*_d_ values. Some antagonists were wrongly present as agonists in the database. These discrepancies were corrected together with some wrong affinity data. This finally resulted in two sets containing 118 agonists and 115 antagonists.

For the decoys, the enhanced directory of useful decoys (DUD-E) [36] was downloaded for the AR. From this directory, we picked a diverse subset of 3000 compounds to get a ratio of actives to decoys of about 1:30. The diverse set was created by clustering with the SkeletonSpheres Descriptor implemented in the DataWarrior package [41].

### 3.2. Preparation of Experimental AR Structures for Ensemble Docking

All androgen WT crystal structures with a resolution of better or equal than 2.5 Å were downloaded from the protein data bank (PDB, www.rcsb.org). The structures were refined with the Protein Preparation Wizard [42,43] from the Small-Molecule Drug Discovery Suite from Schrödinger. All hydrogens were added and protonation states were determined for a pH of 7.4. Missing loops and side-chains were added and the hydrogen network was refined, followed by a short minimization. We clustered the structures according to their binding site similarity, as described elsewhere [44]. We identified five clusters from the hierarchical clustering and the centroids of these clusters composed the ensemble used for the docking.

### 3.3. Molecular Dynamics Simulations

Receptor-ligand complexes of AR antagonists bound to mutants and AR WT as well as AR agonists bound to AR WT were refined with the Protein Preparation Wizard [43] (see previous paragraph). All molecular dynamics simulations were carried out with Desmond [45,46]. The default relaxation protocol was applied, consisting of the following steps: (i) 100 ps Brownian Dynamics in the NVT ensemble at 10 K and solute heavy atoms restraints (50 kcal/mol/Å^2^) (ii) 12 ps simulation in the NVT ensemble, keeping the restraints and temperature at 10 K (iii) 12 ps simulation in the NPT ensemble, keeping restraints and temperature at 10 K (iv) 12 ps simulation in the NPT simulation with solute heavy atom restraints at 300 K and (v) 24 ps simulation in the NPT ensemble at 300 K without restraints. Production runs were performed in the NPT ensemble at 300 K and 1.01 bar for 100 ns. The TIP4P-EW water model was used and counterions were added to neutralize the net charge of the system. The temperature and pressure were controlled using a Langevin thermostat (relaxation time 1.0 ps) and a Langevin barostat (relaxation time 2.0 ps). Electrostatic interactions were treated using the particle mesh Ewald summation. The OPLS3 force field was employed [47].

Representative structures for each trajectory were obtained by an affinity propagation clustering based on the heavy atom RMSD values of all heavy atoms within 5.0 Å of the ligand. The centroids of the three most populated clusters were taken as the representative structures.

### 3.4. Binding Pose Metadynamics

Metadynamics facilitates enhanced sampling for Molecular Dynamics simulations by penalizing already visited configurational states of the system, whereby a bias is added set of collective variables (CVs) [48]. Binding Pose Metadynamics [40] is a tool developed by Schrödinger aiming to identify the most stable (and therefore considered best) pose from an induced-fit docking run. The selected collective variable is the RMSD (root mean square deviation) of the ligand atoms and a selected set of binding site atoms from an initial, equilibrated configuration. A set of short metadynamics simulations is run and the free energy of the system is evaluated as a function of the CV. The pose stability is expressed as the thermodynamically most favorable RMSD from the starting configuration. If this RMSD is low, the initial pose can be regarded as stable. The protocol uses the Desmond engine [45] and the OPLS3 force field [47].

### 3.5. Glide Docking

Flexible-ligand rigid-protein docking was performed using the Glide program from Schrödinger [49]. The applied scoring function was Glide SP (standard precision). The ligand structures were prepared using LigPrep [50]. Protonation states were generated at pH 7.0 ± 2.0, and a state penalty was added to the final docking score. A detailed list of the applied settings for the docking is given in the Supporting Information (S2).

### 3.6. Induced-Fit Docking (IFD)

IFD was performed using the induced-fit docking protocol from Schrödinger [51]. The detailed settings are tabulated in the supporting information (S3).

### 3.7. Ensemble Docking

Ensemble docking usually involves flexible ligand—fixed protein docking towards a representative ensemble of protein structures [31,32,34,52,53]. This approach accounts for the protein-flexibility at a moderate computational cost. In our implementation, all ligands where docked to all members of the ensemble and the best Glide-SP score of a ligand across the ensemble was taken as the final docking score. The successful outcome of an ensemble docking depends on the generation and selection of the structural ensemble [34]. Docking to the ensemble should give accurate docking poses [52] and successful discrimination between actives and inactives. In our study, we selected ensembles from a pool of protein conformations generated by MD simulations. We generated a pool of structures from MD simulations of agonists bound to AR and of antagonists bound to AR, which were then used to construct agonist and antagonist ensembles respectively. We chose an ensemble size of five and selected the best ensembles depending on the objective function (successful discrimination of actives and inactives). We chose an exhaustive search procedure for picking the best ensemble, meaning that all possible combinations of five structures were evaluated from the pool.

### 3.8. Evaluation of the Docking Performance

The chosen metric for the docking performance was the ROC AUC, the area under the curve of the receiver operator characteristic curve. For docking and scoring, the AUC gives the probability that a randomly chosen true active is ranked higher than a randomly chosen decoy. A ROC AUC of 1.0 would thereby indicate that all actives are ranked before the inactives/decoys. The ROC AUC metric was criticized for an insufficient incorporation of early enrichment [54]. However it has assets such as statistical reliability [55] and robust behavior towards decoys that are actually active against the target [55] and is in general a widely applied metric for the assessment of docking performance. In addition, since we also look at androgen binding from the point of view of toxicology, where early enrichment is not a main objective, and not only virtual screening, we regarded the ROC AUC as a good measure for the docking performance at the AR. In general, the enrichment obtained by a docking and scoring approach is evaluated by the ability of the methodology to discriminate actives from inactives. For the AR, actives can be furthermore divided into agonist and antagonists. An accurate method would successfully distinguish agonists from decoys, antagonists from decoys and agonists from antagonists.

## 4. Conclusions

Molecular Dynamics simulations in combination with molecular docking was applied to study the ability of structure-based screening methods to predict AR agonism and antagonism. Docking against an ensemble derived from experimental crystal structures of AR in agonist conformation was shown to yield good enrichments for the screening of agonists, but is insufficient for antagonist docking. This was circumvented by using a workflow that combines induced-fit docking of known antagonists towards the AR, assessing the most stable poses and generate structural ensembles by running 100 ns MD simulations from these structures followed by a clustering procedure. To our knowledge, this the first study that benchmarks a docking approach at the AR aiming at prediction of agonism and antagonism.

This MD-derived ensemble of antagonist-like protein conformations was shown to yield superior docking performance in terms of discriminating antagonists from decoys, compared to the ensemble from the crystal structure.

Furthermore, an ensemble of agonist-like protein conformations was created by running MD simulations of bound agonists. This ensemble also showed better enrichments than the ensemble from the experimental crystal structures. A remaining challenge is the discrimination between agonists and antagonists. The antagonist ensemble shows high promiscuity towards both agonists and antagonists, whereas the agonist ensemble is selective for agonists. As a result, screening selectively for agonists is possible, by docking the library only towards the agonist set and taking the highest-scoring compounds as leads. However, selectively screening for antagonists based only on docking is not feasible.

Lagarde et al. recently showed that nuclear receptor agonists and antagonists can be discriminated using a 3D pharmacophore approach [56]. Therefore, substituting the SBVS with a ligand-based method can be expected to yield good results for a selective screening of antagonists. Furthermore, studies employing long-timescale MD simulations showed different protein structural dynamics for agonist-bound AR compared to antagonist-bound AR [14,21] and differing protein-ligand interactions for agonist and antagonists. A hierarchical approach, combining pharmacophore screening, molecular docking, and MD simulations for the best-scoring ligands from the docking can expected to lead to a focused screening towards AR agonists and antagonists. Such studies can also be extended to different AR mutations for the screening for AR pan-antagonists.

We think that this study improves the potential of SBVS and structure-based design at the androgen receptor and leads to more reliable toxicology prediction of environmental chemicals by better accounting for AR antagonist. We could successfully create an ensemble of antagonist-like AR structures that can accommodate AR antagonists. These structures are made publicly available (see Supporting Information) and are aimed to help future developments of AR antagonists and more confident prediction of AR disrupting chemicals.

We furthermore showed the potential of MD simulations to generate meaningful protein conformations with desired objectives in the absence of experimental crystal structures.

To promote further use of our findings, a link for downloading the agonist ensemble, antagonist ensemble and ligand sets is provided in the Supporting Information (S1).

## Figures and Tables

**Table 1:** Results from the cross-docking experiment. Listed are the PDB IDs of the ligands and corresponding protein structures together with their Glide-Scores.

Protein Structure	3B66	3G0W	3V49	4HLW	2PNU
ligand					
3B66	−12.2	−8.5	−8.9	N/A	−10.9
3G0W	−10.8	−11.2	−10.6	−8.4	−10.0
3V49	−10.5	N/A	−12.2	N/A	−9.7
4HLW	−8.8	N/A	−9.4	−11.3	−10.0
2PNU	N/A	N/A	N/A	N/A	−14.2

**Table 2:** Docking performances of the different ensembles against various sets of actives and decoys.

Ensemble	ROC AUC	Actives	Decoys
crystal structures	0.92	agonists	DUD-E
crystal structures	0.80	agonists	antagonists
crystal structures	0.65	antagonists	DUD-E
MD_antagonist_1	0.86	antagonists	DUD-E
MD_antagonist_1	0.29	antagonists	agonists
MD_antagonist_1	0.92	agonists	DUD-E
MD_agonist_1	0.96	agonists	DUD-E
MD_agonist_1	0.82	agonists	antagonists
MD_agonist_1	0.69	antagonists	decoys
MD_antagonist_2	0.96	agonists	decoys
MD_agonist_2	0.74	antagonists	decoys
MD_antagonist_3	0.79	antagonists	decoys
MD_antagonist_3	0.56	antagonists	agonists

## Supplementary Materials

Supplementary materials can be found at http://www.mdpi.com/1422-0067/19/6/1784/s1.
