# Supplementary material for: Endocrine Disruption at the Androgen Receptor: Employing Molecular Dynamics and Docking for Improved Virtual Screening and Toxicity Prediction
Source: Int J Mol Sci. 2018 Jun 15;19(6):1784. doi: 10.3390/ijms19061784 (PMC6032383; doi:10.3390/ijms19061784)
Supplement: Supplementary file 1 [file ijms-19-01784-s001.pdf]

Supporting Information

S1. Link to download the MD_antagonist_1 and MD_agonist_1 ensembles for ensemble docking together with the set of agonists, antagonists and decoys for benchmarking of the docking performance:

https://figshare.com/articles/Androgen_receptor_ensemble_docking/6170159

S2. Parameters for Glide_SP docking

```
[STAGE:LIGPREP]
   STAGECLASS   ligprep.LigPrepStage
   INPUTS   ORIGINAL_LIGANDS,
   OUTPUTS   LIGPREP_OUT,
   RECOMBINE   YES
   RETITLE   YES
   MIXLIGS   YES
   SKIP_BAD_LIGANDS   YES
   UNIQUEFIELD   NONE
   OUTCOMPOUNDFIELD   s_vsw_compound_code
   USE_EPIK   YES
   METAL_BINDING   NO
   PH   7.0
   PHT   2.0
   NRINGCONFS   10
   COMBINEOUTS   NO
   STEREO_SOURCE   geometry
   NUM_STEREOISOMERS   32
   REGULARIZE   NO

[STAGE:SAMPLERINGS]
   STAGECLASS   macromodel.SampleRingsStage
   INPUTS   LIGPREP_OUT,
   OUTPUTS   SAMPLERINGS_OUT,
   OUTCONFS_PER_SEARCH   10

[STAGE:POSTLIGPREP]
   STAGECLASS   ligprep.PostLigPrepStage
   INPUTS   SAMPLERINGS_OUT,
   OUTPUTS   POSTLIGPREP_OUT,
   UNIQUEFIELD   s_vsw_compound_code
   OUTVARIANTFIELD   s_vsw_variant
   PRESERVE_NJOBS   YES
   LIMIT_STEREOISOMERS   YES
   MAXSTEREO   4
   REMOVE_PENALIZED_STATES   YES

[STAGE:PRE_DOCK_SP_1]
```

```
   STAGECLASS   gencodes.RecombineStage
   INPUTS   POSTLIGPREP_OUT,
   OUTPUTS   DOCK_SP_1_INPUT,
   NUMOUT   njobs
   OUTFORMAT   maegz
   MIN_SUBJOB_STS   300
   MAX_SUBJOB_STS   3000
   GENCODES   NO
   UNIQUEFIELD   s_vsw_compound_code

[STAGE:DOCK_SP_1]
   STAGECLASS   glide.DockingStage
   INPUTS   DOCK_SP_1_INPUT, GRID_1
   OUTPUTS   SP_OUT_1,
   RECOMBINE   NO
   PRECISION   SP
   UNIQUEFIELD   s_vsw_compound_code
   PERCENT_TO_KEEP   100
   DOCKING_METHOD   confgen
   POSES_PER_LIG   1
   WRITE_XP_DESC   NO
   NENHANCED_SAMPLING   1
   BEST_BY_TITLE   YES
   LIG_VSCALE   0.8
   LIG_CCUT   0.15
   MAXATOMS   300
   MAXROTBONDS   50
   AMIDE_MODE   penal
   POSE_OUTTYPE   PV
   POSTDOCK   YES
   POSTDOCKSTRAIN   YES
   COMPRESS_POSES   YES
   EPIK_PENALTIES   NO
   FORCEPLANAR   YES
```

S3. Parameters for Induced Fit docking

```
TAGE GLIDE_DOCKING2
  BINDING_SITE ligand A:1
  INNERBOX 10.0
  OUTERBOX auto
  LIGAND_FILE  InducedFit__2oz7.mae
  LIGANDS_TO_DOCK all
  GRIDGEN_RECEP_CCUT  0.25
  GRIDGEN_RECEP_VSCALE 0.50
  DOCKING_PRECISION SP
  DOCKING_LIG_CCUT  0.15
  DOCKING_CV_CUTOFF  100.0
  DOCKING_LIG_VSCALE 0.50
  DOCKING_POSES_PER_LIG 20
```

```
  DOCKING_RINGCONFCUT 2.5
  DOCKING_AMIDE_MODE penal

STAGE COMPILE_RESIDUE_LIST
  DISTANCE_CUTOFF         5.0

STAGE PRIME_REFINEMENT
  NUMBER_OF_PASSES      1
  USE_MEMBRANE no
  OPLS_VERSION OPLS3

STAGE SORT_AND_FILTER
  POSE_FILTER         r_psp_Prime_Energy
  POSE_KEEP30.0

STAGE SORT_AND_FILTER
  POSE_FILTER         r_psp_Prime_Energy
  POSE_KEEP20#

STAGE GLIDE_DOCKING2
  BINDING_SITE ligand Z:999
  INNERBOX 10.0
  OUTERBOX auto
  LIGAND_FILE  InducedFit__2oz7.mae
  LIGANDS_TO_DOCK self
  GRIDGEN_RECEP_CCUT  0.25
  GRIDGEN_RECEP_VSCALE 1.00
  DOCKING_PRECISION SP
  DOCKING_LIG_CCUT  0.15
  DOCKING_CV_CUTOFF  0.0
  DOCKING_LIG_VSCALE 0.80
  DOCKING_POSES_PER_LIG 1
  DOCKING_RINGCONFCUT 2.5
  DOCKING_AMIDE_MODE penal

STAGE SCORING
  SCORE_NAME  r_psp_IFDScore
  TERM 1.0,r_i_glide_gscore,0
  TERM 0.05,r_psp_Prime_Energy,1
  REPORT_FILE report.csv
```
